# Cajal and the discovery of the Golgi method: a neuroanatomist’s dream

**DOI:** 10.1007/s12565-025-00840-7

**Published:** 2025-05-05

**Authors:** Javier DeFelipe

**Affiliations:** 1 Laboratorio Cajal de Circuitos Corticales, Centro de Tecnología Biomédica, Universidad Politécnica de Madrid, Campus Montegancedo S/N, 28223 Pozuelo de Alarcón, Madrid Spain; 2 Instituto Cajal, CSIC, Avenida Doctor Arce, 37, 28002 Madrid, Spain; 3 Centro de Investigación Biomédica en Red Sobre Enfermedades Neurodegenerativas (CIBERNED), ISCIII, Madrid, Spain

**Keywords:** Histological techniques, Golgi method, Neural connections, Neuron theory, Reticular theory, Dynamic polarization, Neuroscience history

## Abstract

This article explores the historical progression of studying neuronal connections, beginning with nineteenth-century advancements in light microscopy and histological techniques. Early methods were limited in terms of their capacity to trace neuronal connections, but a breakthrough came with Camillo Golgi’s “black reaction” staining method, later refined and extensively used by Santiago Ramón y Cajal. Cajal’s observations supported the Neuron Theory, which proposed that neurons communicate via specialized points of contact, contradicting the prevailing Reticular Theory of a continuous neural network, which was supported by Golgi. This contrast is particularly intriguing because, although Golgi and Cajal used the same black reaction technique and similar microscopes, their interpretations of the microscopic world diverged significantly. An important consequence of the Neuron Theory was Cajal’s Law of Dynamic Polarization, which proposed that neurons generally consist of three functionally distinct regions: a receptor apparatus (dendrites and soma), an emission apparatus (axon), and a distribution apparatus (terminal axonal arborization). He applied this principle across various parts of the nervous system and to different neuron types, enabling the generation of the first detailed circuit diagrams of the brain. Cajal’s observations, concepts, and theories had a profound impact—not only on researchers of his time, but also on modern neuroscience. This article reflects on the early studies of neuronal connections, highlighting the scientific climate in which Golgi and Cajal initiated their groundbreaking research.

**Supplementary Information:**

The online version contains supplementary material available at 10.1007/s12565-025-00840-7.

## Introduction

The story of the detailed study of the connections between neurons began in the nineteenth century, driven by advancements in light microscopy and histological techniques for visualizing the microscopic world of the brain. However, early attempts to understand the structure and function of the nervous system were severely hindered by the limitations of the histological techniques that were available at the time. These techniques only allowed the observation of the cell body and proximal portions of dendrites and axons, making it impossible to trace the trajectories of thin axons or visualize their terminal arbors. Thus, the capacity to trace the connections between neurons—a crucial aspect if the nervous system were to be understood—remained technically elusive. Santiago Ramón y Cajal (1852–1934) articulated this beautifully in *Recuerdos de mi vida* (Cajal, 1917):

The great enigma in the organization of the brain revolves around our need to ascertain how the nervous ramifications end and how neurons are mutually connected. Referring to a simile already mentioned, the idea was to inquire how the roots and branches of the trees in the grey matter terminate, so that in such a dense jungle, in which there are no gaps thanks to its refined complexity, the trunks, branches and leaves touch everywhere.

In this article, I will examine a pivotal moment in the history of neuroscience: the discovery of the staining method known as the *reazione nera* (“black reaction”) by Camillo Golgi (1843–1926), later named the Golgi method, and its subsequent application by Cajal to study the brain. In particular, I will discuss Cajal’s interpretation of the connections between neurons, supporting the idea that impulses traversed from one cell to another via specialized points of contact or contiguity (Neuron Theory), rather than by continuity (Reticular Theory). It is remarkable to consider how the way in which scientists work has evolved over the relatively short period of the last 150 years. We have shifted from working in individual laboratories to a more efficient and powerful interdisciplinary approach, collaborating with other laboratories, and, above all, utilizing new sophisticated techniques and microscopes. An obvious example —in the context of connections between neurons—is the introduction of electron microscopy in the 1950s, along with the development of new methods to prepare nervous tissue for fine structural analysis (for example, fixation in osmium tetroxide and/or aldehydes, epoxy embedding, etc.:Palade and Palay 1954; De Robertis and Bennett 1955; Palay 1956; De Robertis 1959; Gray 1959a, b). This novel technology allowed the examination of the ultrastructure of the *synapse*, a term coined by the renowned neurophysiologist Charles Sherrington (1857–1952) in 1897 (Foster and Sherrington, 1897), for the hypothetical one-way contact between axon terminals and somata or dendrites. He wrote the following on this subject, offering a fascinating glimpse into the major challenge and importance of histology in understanding the functional and structural organization of the nervous system (Sherrington 1947):

As to the existence or non-existence of a surface of separation or membrane between neurone and neurone, that is a structural question on which histology might be competent to give valuable information. In certain cases, especially in Invertebrata, observation (Apáthy, Bethe, etc.) indicates that many nerve-cells are actually continuous one with another. It is noteworthy that in several of these cases the irreversibility of direction of conduction which is characteristic of spinal reflex-arcs is not demonstrable […]. But in the neurone-chains on the gray-centred system of vertebrates, histology on the whole furnishes evidence that a surface of separation does exist between neurone and neurone. […] It seems therefore likely that the nexus between neurone and neurone in the reflex-arc, at least in the spinal arc of the vertebrate, involves a surface of separation between neurone and neurone; and this as a transverse membrane across the conductor must be an important element in intercellular conduction. […] In view, therefore, of the probable importance physiologically of this mode of nexus between neurone and neurone, it is convenient to have a term for it. The term introduced has been *synapse* (Foster and Sherrington 1897).

The advent of electron microscopy immediately enabled the confirmation of a fundamental principle of the neural theory, namely that presynaptic and postsynaptic elements are physically separated by a space of approximately 10 to 20 nm, known as the “synaptic cleft” (reviewed in Peters et al. 1991). The introduction of the combined Golgi-electron microscope technique in the 1970s and 1980s spurred the development of methods for examining light-microscopically identified cells under electron microscopy. This paved the way for reconstructing complex neural circuits (reviewed in Blackstad 1970, 1981; Freund and Somogyi 1989; Fairén, 2005). Since then, advancements in light and electron microscopy—including immunocytochemistry, combined tract-tracing and histochemical/immunocytochemical methods, intracellular dye injections in physiologically characterized neurons, and genetic circuit-tracing approaches—have significantly propelled the study of these circuits. It is interesting to ponder what scientists of Golgi and Cajal’s era would have thought upon witnessing such remarkable progress in such a short space of time that would undoubtedly have surpassed the imagination of those great early neuroanatomists.

The present work is largely based on several of my previously published works on Cajal’s contributions to the detailed study of the nervous system, particularly *Cajal’s neuronal forest: science and art* (DeFelipe 2018).

## The beginning

**Fig. 1:**
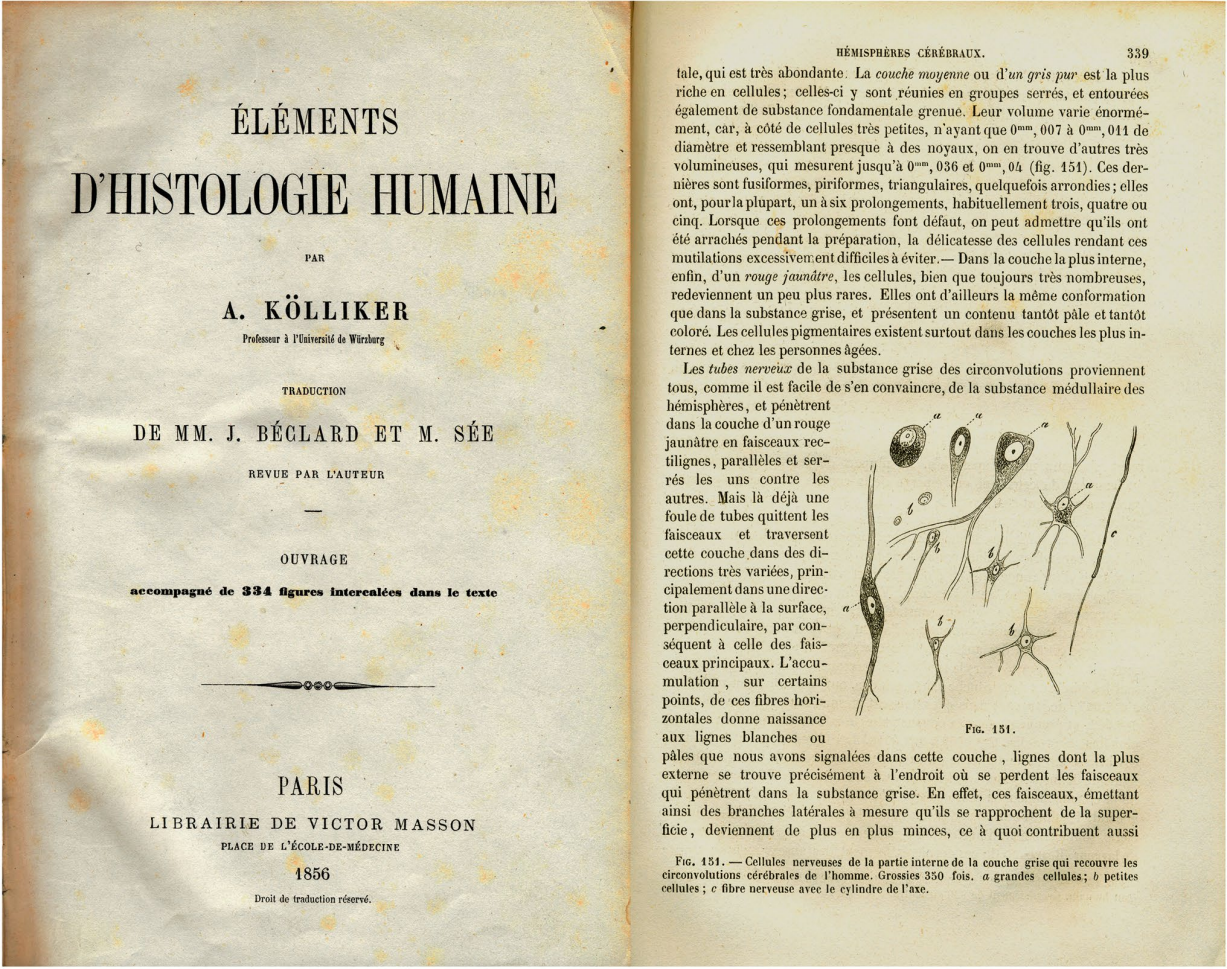
*Left*, cover of the French edition (Kölliker, 1956) of Kölliker’s classic book *Handbuch der Gewebelehre des Menschen* (Kölliker, 1852). *Right*, Fig. 151 of the book where various morphological types of nerve cells in the human cerebral cortex are illustrated

Before the arrival of the Golgi method, very little was known about the microscopic structure of the nervous system. A good example can be seen in Fig. 1, taken from the classic book by the eminent scientist Rudolf Albert von Kölliker (1817–1905), published in 1852—the year Cajal was born. In this figure, the morphology of cortical neurons is depicted in a very simplistic manner.

**Fig. 2:**
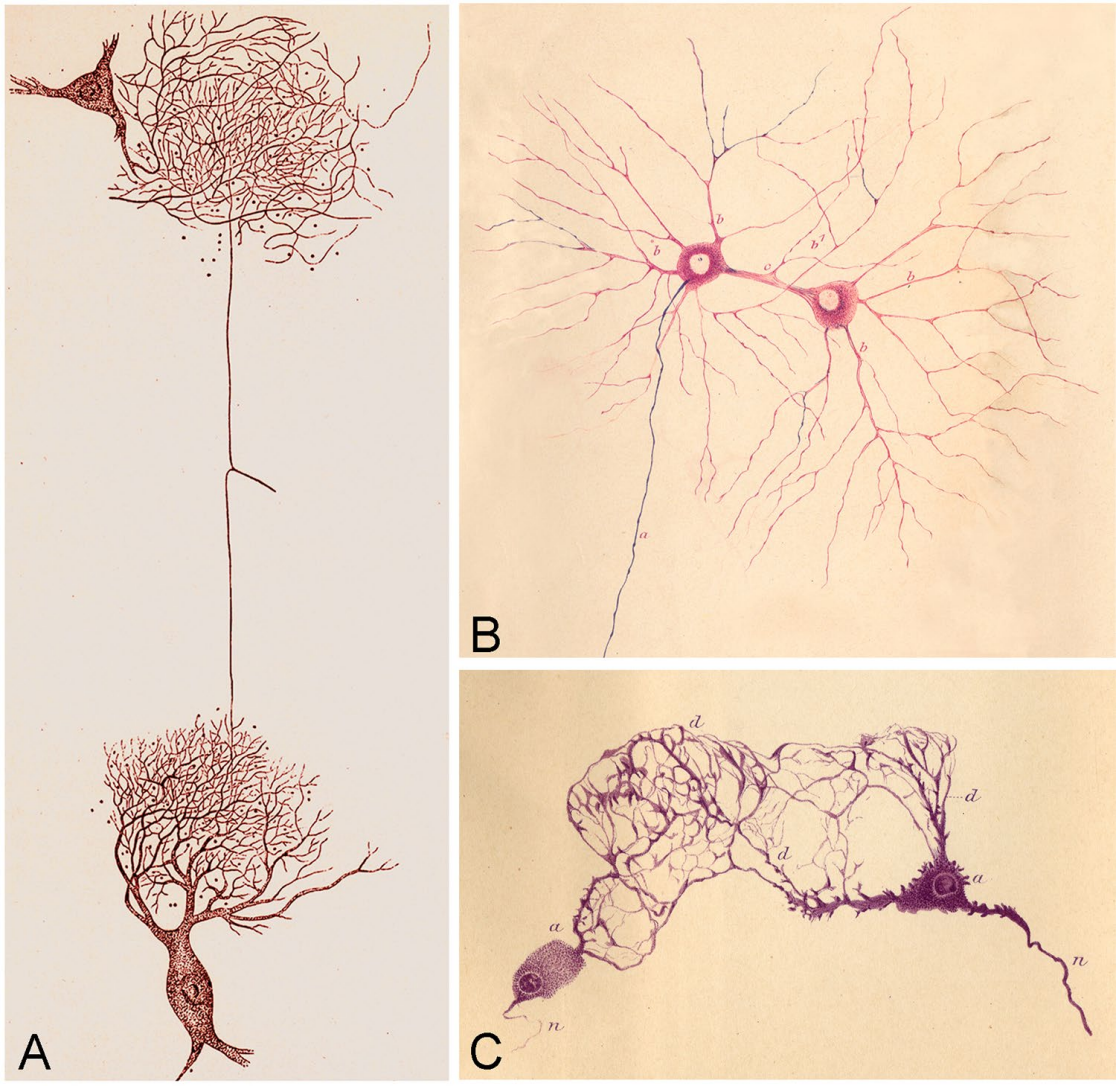
Reticular Theory. (**A**) Drawing by Gerlach (1872) showing two nerve cells from the spinal cord of the ox (prepared with carmine and ammonia). According to this author, the conduction of nervous activity takes place through a network of neural elements formed by dendrites (dendritic network) and axons (axonal network). (**B**, **C**) Drawings of ganglion cells of the human retina and cells of the dog gallbladder ganglia drawn by Dogiel in 1893 and 1899, respectively (Dogiel, 1893, 1899).

**Fig. 3:**
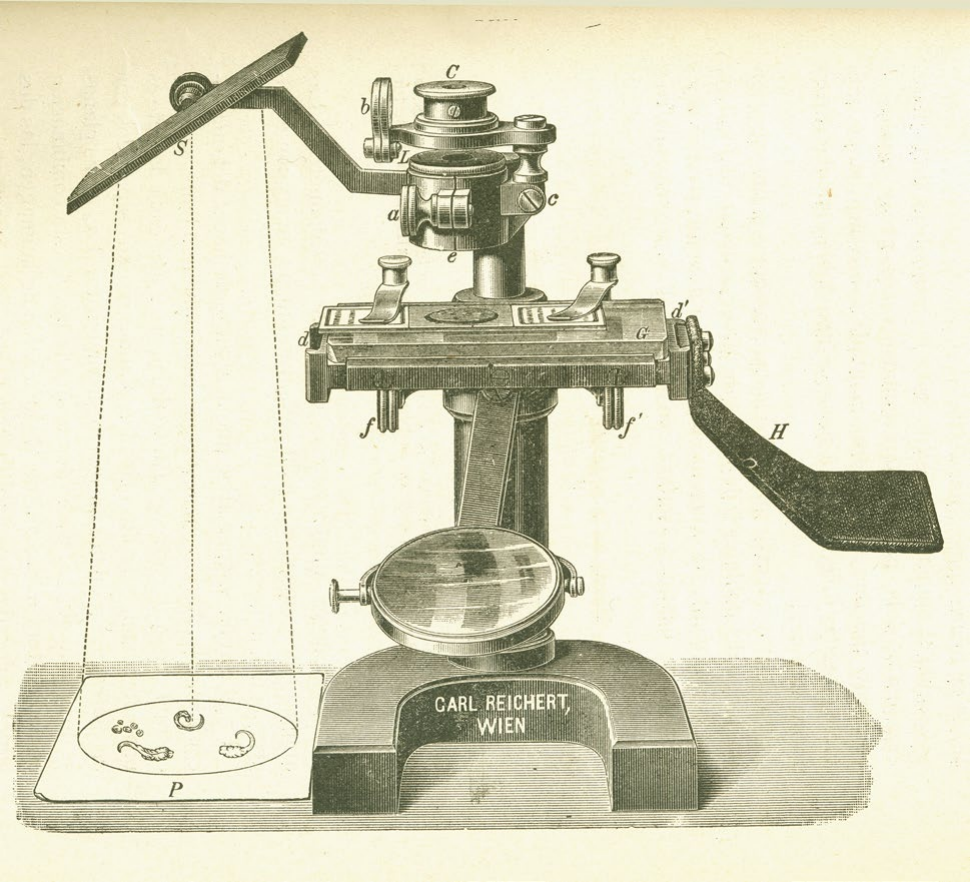
Reichert microscope, with a camera lucida of the Abbe type, published by Cajal in his book *Manual de histología normal y de técnica micrográfica* (Cajal 1914)

At that time, the prevailing view regarding the organization of the nervous system was the Reticular Theory. This theory was primarily developed by Joseph von Gerlach (1820–1896), who is considered its founding figure (Gerlach 1872). Gerlach proposed that the conduction of nervous activity occurs through a network of neural elements formed by dendrites (dendritic network) and axons (axonal network) (Fig. 2A). According to this theory, axons had two origins: direct (from the cell body) and indirect (via the dendritic network). Consequently, the connections between nerve cells were considered to be dual, occurring both through the main axon and via the dendritic network, which itself was part of the axonal network. Figure 2B and C shows drawings by Aleksander Dogiel (1852–1922) that support the Reticular Theory. Figure 2B illustrates two neurons connected through a thick common dendrite (“inter-protoplasmic bridges”), rather than existing as independent elements. This figure is particularly interesting because it was produced with the aid of a camera lucida, an optical device used to assist in accurately tracing or drawing images observed under a microscope (Fig. 3). The device works by utilizing a mirror to superimpose the image of an object onto a drawing surface, such as a sheet of paper. This allows the user to view both the object and their hand simultaneously, facilitating the reproduction of the object’s shape and details. However, this drawing by Dogiel serves as a good example demonstrating that the use of this device did not prevent the observer from making incorrect observations.

It was in this scientific climate that a significant advancement in neuroscience occurred in 1873 with the introduction of Golgi’s black reaction staining method. This groundbreaking technique made it possible to observe neurons and glial cells, along with all their parts, and—for the first time—enabled the tracing of connections between neurons. On February 16, 1873, Golgi wrote the following letter to his friend Niccolò Manfredi (Mazzarello 1999):

I spend long hours at the microscope. I am delighted that I have found a new reaction to demonstrate even to the blind the structure of the interstitial stroma of the cerebral cortex. I let the silver nitrate react with pieces of brain hardened in potassium dichromate. I have obtained magnificent results and hope to do even better in the future

In this letter, Golgi referred to a new staining technique for the nervous system that labeled neurons and glial cells black, the “*reazione nera*”. This method was first published in the *Gazzeta Medica Italiani* on August 2, 1873 (Golgi 1873) in an article entitled *Sulla struttura della sostanza grigia del cervello* (“On the Structure of the grey substance of the cerebrum”). In this article, Golgi wrote:

Taking advantage of the method, found by me, to stain black the elements of the brain […] I happened to discover some facts concerning the structure of the cerebral grey matter that I believe merit immediate communication.

**Fig. 4:**
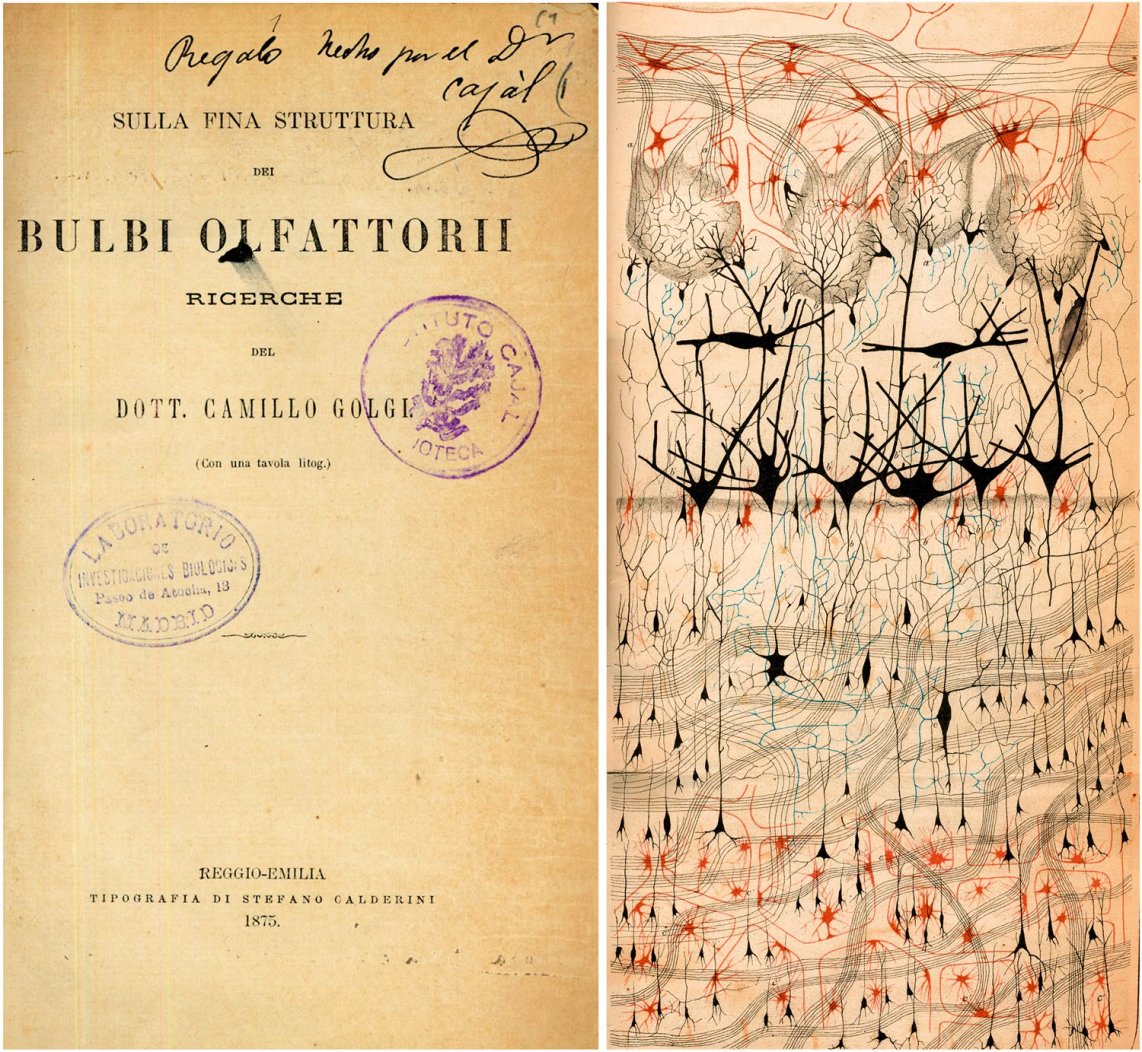
The first illustration by Golgi of a Golgi-impregnated preparation of the nervous system. “Semi-schematic drawing of a fragment of a vertical section of the olfactory bulb of a dog”. Taken from Golgi (1875). This figure beautifully illustrates the cytoarchitecture of the olfactory bulb stained using the Golgi method, with different cell types clearly identified (DeFelipe 2002a)

**Fig. 5:**
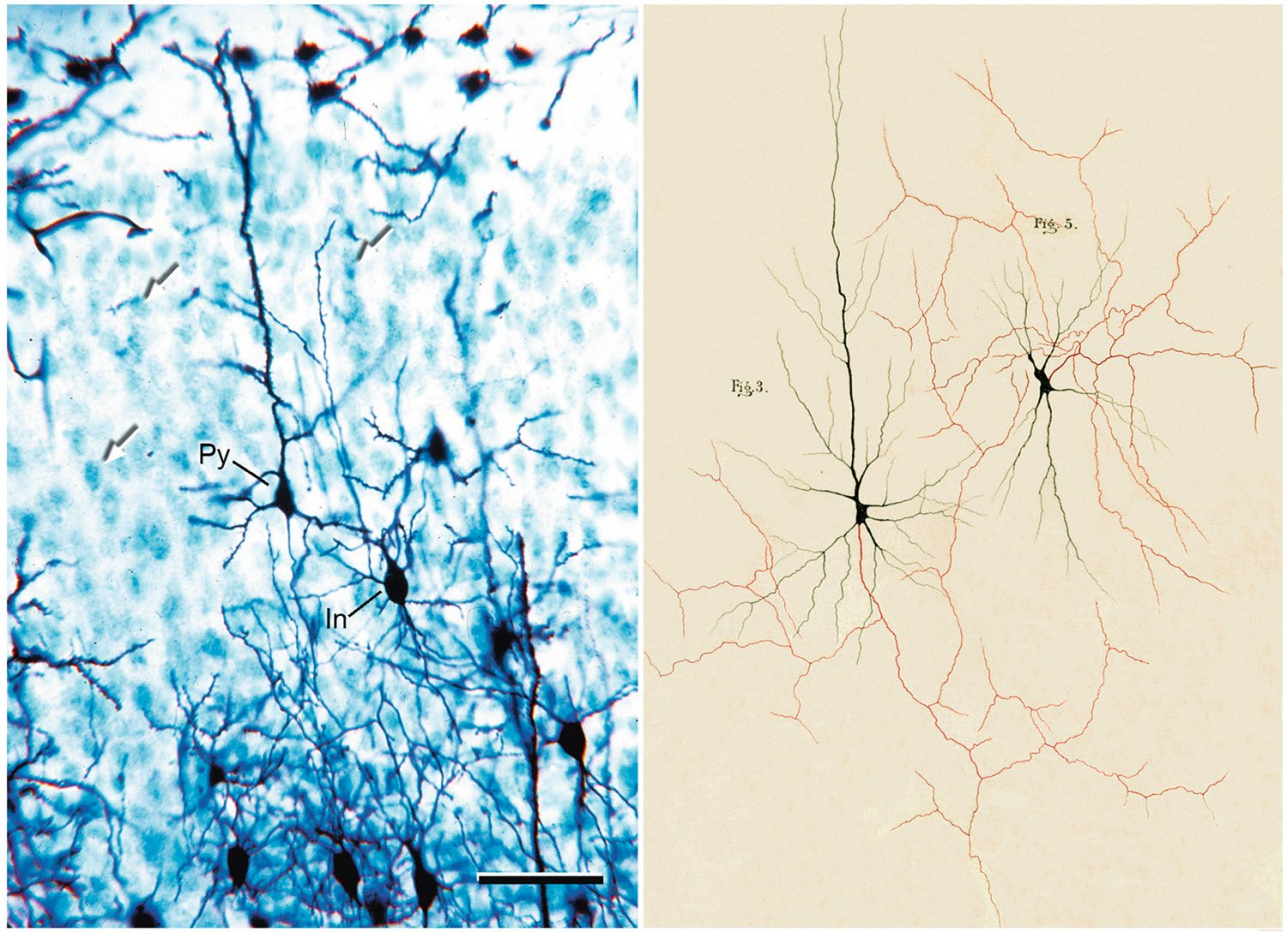
*Left*, Preparation of mouse cerebral cortex stained with the Golgi method and counterstained with the method of Nissl. With the method of Golgi, only the cell bodies and processes of a small number of cells are stained black, while with the method of Nissl, all cell bodies are stained blue (white arrows indicate some stained cells). Py, pyramidal cell; In, interneuron**.** Scale bar: 100 µm. *Right*, Drawing taken from Plate XI from Golgi (1882−1883), illustrating different types of neurons stained using the Golgi method, “specifically intended to show the origin and branching of the axon”. The axon is highlighted in red. According to the legend, the cell labeled *Fig. 3* represents “a large ganglionic cell [pyramidal neuron] from the middle layer of the human motor cortex. Its axon, although giving off numerous axonal collaterals, remains a well-defined fiber extending into the [white matter]”. The cell labeled *Fig. 5* represents a nerve cell from the superficial gray layer of the superior colliculus of the cat. As stated in the figure, “its axon, emerging from one side of the cell body, soon broke up into an innumerable series of fine fibrils, which, continuing to subdivide, predominantly extended toward the free surface of the superior colliculus.” This neuron would be a short-axon cell or interneuron. See below for further explanation

The staining protocol was relatively simple, requiring the “prolonged immersion of the tissue, previously hardened with potassium or ammonium dichromate, in a 0.50 or 1.0% solution of silver nitrate.” This technique enabled the visualization of neurons and glia in histological preparations for the first time, revealing their full structure: the cell body, dendrites, and axon in neurons, and the cell body and processes in glial cells (Fig. 4). Another important advantage of the Golgi method was that only a small proportion of the neurons in a given preparation were stained, permitting individual neurons to be examined in great morphological detail. This made it possible to (i) characterize and classify neurons, (ii) study their connections by following the trajectory of their axons, and (iii) identify their possible contacts with other neurons (Fig. 5).

## Cajal arrives on the scene in 1888

**Fig. 6:**
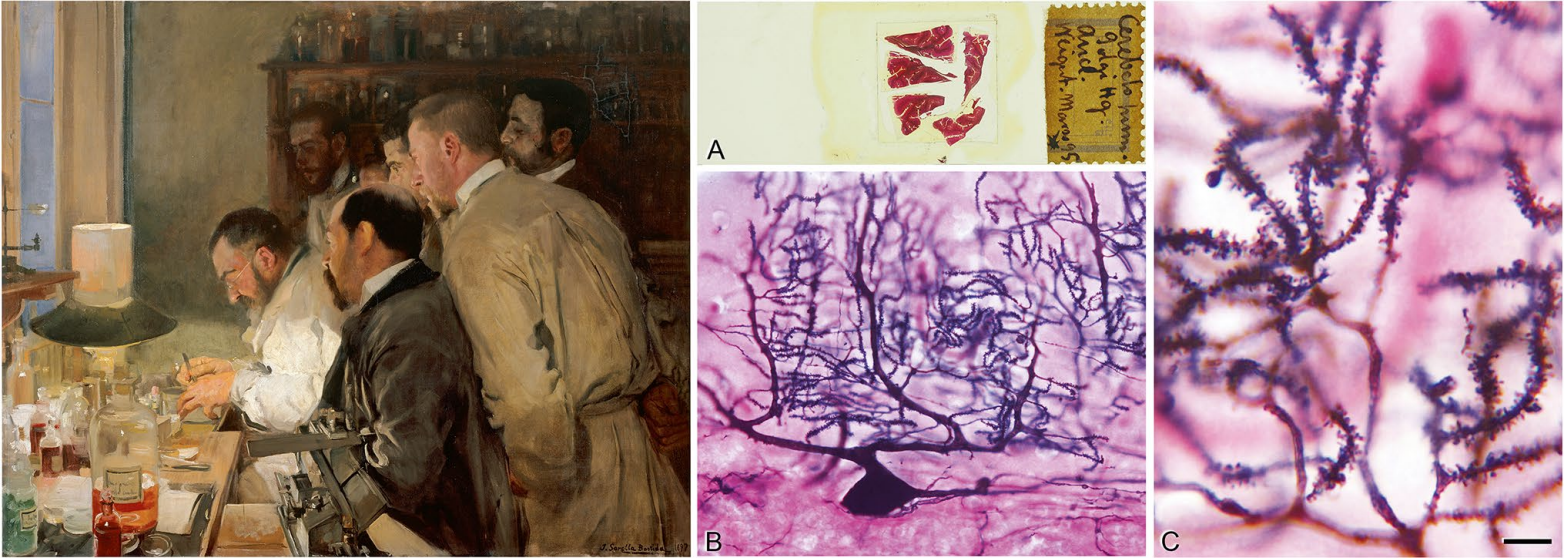
*Left*: *Una investigación o el Dr. Simarro en el laboratorio* (1897) (An investigation or Dr. Simarro in the laboratory), by the painter Joaquin Sorolla Bastida (1863–1923), showing Luis Simarro (white coat) working in his laboratory. The large glass jar that appears in the foreground contains potassium dichromate, which —together with silver nitrate—make up the two main ingredients of the Golgi method. Madrid, Museo Sorolla (Inv. 417). *Right*: **A**, Photograph of a preparation from Simarro of the human cerebellum stained with the Golgi method in combination with the Weigert method. **B**, Low power photomicrograph showing a typical Purkinje cell. **C**, Higher magnification of **B** showing dendritic spines. Scale bar shown in C indicates 30 µm in B and 4.5 µm in **C**. The histological images (Legado Simarro, Universidad Complutense, Madrid) were taken by Iñigo Azcoitia and Alberto Muñoz (Universidad Complutense). The figure was modified from DeFelipe (2018)

In 1887, a fortuitous encounter in Madrid with Luis Simarro (1851–1921) marked a milestone in the early development of Cajal’s scientific career. Simarro, a renowned psychiatrist and neurologist with a keen interest in histology, showed Cajal a preparation of nerve tissue stained with the Golgi method (Fig. 6) during a visit to his private laboratory. As we will see later, this method became, in Cajal’s hands, the fundamental tool that allowed him to change the course of the history of neuroscience, marking the birth of modern neuroscience.

**Fig. 7:**
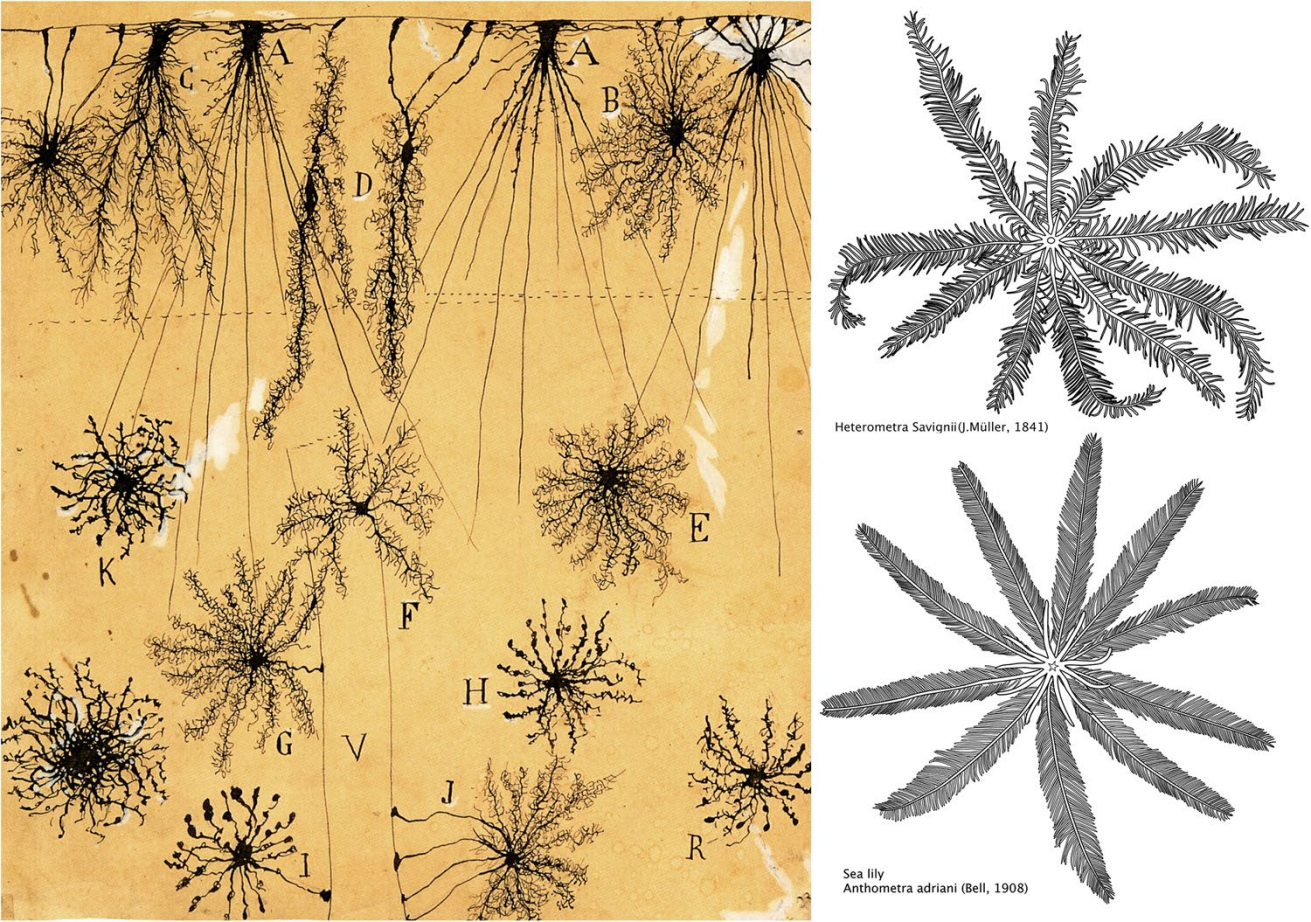
*Left*, Cajal’s drawing of Golgi-impregnated neuroglia. The figure legend states: “Neuroglia of the superficial layers of the cerebrum: child of two months. Golgi Method. *A, B, [C], D*, neuroglial cells of the plexiform layer; *E, F, [G, H, K], R*, neuroglial cells of the second and third layers; *V*, blood vessel; *I, J*, neuroglial cells with vascular [pedicles]”. The astrocyte vascular end-feet on blood vessels confirmed the observation made by Golgi in 1885, who noticed that Golgi-impregnated processes from neuroglial cells were frequently in contact with both blood vessels (vascular end-feet or “sucker processes”) and neurons (Golgi, 1885). This finding prompted Golgi to suggest that the main function of glial cells was to supply nutrients to the nerve cells. *Right,* Two representative examples of sea lilies (class: Crinoidea, order: Comatulida): Top, *Alecto savignii* = *Heterometra savignii* (Müller 1841) and bottom, *Anthometra adriani* (Bell, 1908). Original drawings from photographs of natural specimens by Ruth Morona (departamento de Biología Celular, Facultad de Biología, Universidad Complutense de Madrid). Taken from DeFelipe (2014)

The historical moment in which Cajal discovered the properties of the Golgi method (Fig. 7) is exquisitely described in many of his writings, but perhaps the best example of this can be found in the French translation of his work *Textura*, which provides an excellent sample of his usual writing style—lively and enthusiastic (Cajal 1909-1911, volume 1, pages 28–29):

In summary, a method was necessary to selectively stain an element, or at most a small number of elements, that would appear to be isolated among the remaining invisible elements. Could the dream of such a technique truly become reality, in which the microscope becomes a scalpel and histology a fine [tool for] anatomical dissection? A piece of nervous tissue was left hardening for several days in Müller’s pure liquid [potassium dichromate] or in a mixture of this [fixative] with osmic acid. Whether it was the distraction of the histologist or the curiosity of the scientist, the tissue was then immersed in a bath of silver nitrate. The appearance of gleaming needles with shimmering gold reflections soon attracted the attention. The tissue was cut, and the sections were dehydrated, cleared, and then examined [with the microscope]. What an unexpected spectacle! On the perfectly translucent yellow background sparse black filaments appeared that were smooth and thin or thorny and thick, as well as black triangular, stellate or fusiform bodies! One would have thought that they were designs in Chinese ink on transparent Japanese paper. The eye was disconcerted, accustomed as it was to the inextricable network [observed] in the sections stained with carmine and hematoxylin where the indecision of the mind has to be reinforced by its capacity to criticize and interpret. Here everything was simple, clear and unconfused. It was no longer necessary to interpret [microscopically] the findings to verify that the cell has multiple branches covered with ‘frost’, embracing an amazingly large space with their undulations. A slender fiber that originated from the cell elongated over enormous distances and suddenly opened out in a spray of innumerable sprouting fibers. A corpuscle confined to the surface of a ventricle where it sends out a shaft, which is branched at the surface of the [brain], and other cells [appeared] like comatulids or phalangidas. The amazed eye could not be torn away from this contemplation. The technique that had been dreamed of is a reality! The metallic impregnation has unexpectedly achieved this fine dissection. This is the Golgi method! … whose clear and decisive images enable us to cast off the famous net of Gerlach, the [dendritic] arms of Valentin and Wagner, as well as many other fanciful hypotheses.

Interestingly, Cajal knew about the existence of the Golgi method before encountering Simarro. However, as he recounts in *Recuerdos de mi vida* (Cajal, 1917, page 73), he initially dismissed the technique, considering it ineffective:

But, as I mentioned, the admirable method of Golgi was then (1887–1888) unknown to the immense majority of neurologists or was underestimated by the few who had precise information about it. Ranvier’s book, my technical bible of those days, devoted only a few descriptive lines to it, written in an indifferent style. It was evident that the French savant had not tried it. Naturally, the readers of Ranvier, like myself, thought this method to be unworthy to be used.

Cajal was so astonished by the exquisite staining of nerve cells that he questioned why this groundbreaking discovery had not already ignited a scientific revolution. In *Recuerdos de mi vida* (Cajal, 1917, pages 34–35), he remarked:

I owe to Luis Simarro the unforgettable favor of having been shown the first good preparations made by the method of silver chromate which I ever saw, and of his having called my attention to the exceptional importance of the book of the Italian savant devoted to the examination of the fine structure of the gray matter [Golgi, 1885]. […] it was [in 1887] in the house of Dr. Simarro at 41 Arco de Santa María Street [now called Augusto Figueroa, Madrid], that for the first time I had the privilege of viewing sections of the brain stained with the method of Weigert-Pal, and particularly… the sections impregnated by the silver method of the sage of Pavia.

**Fig. 8:**
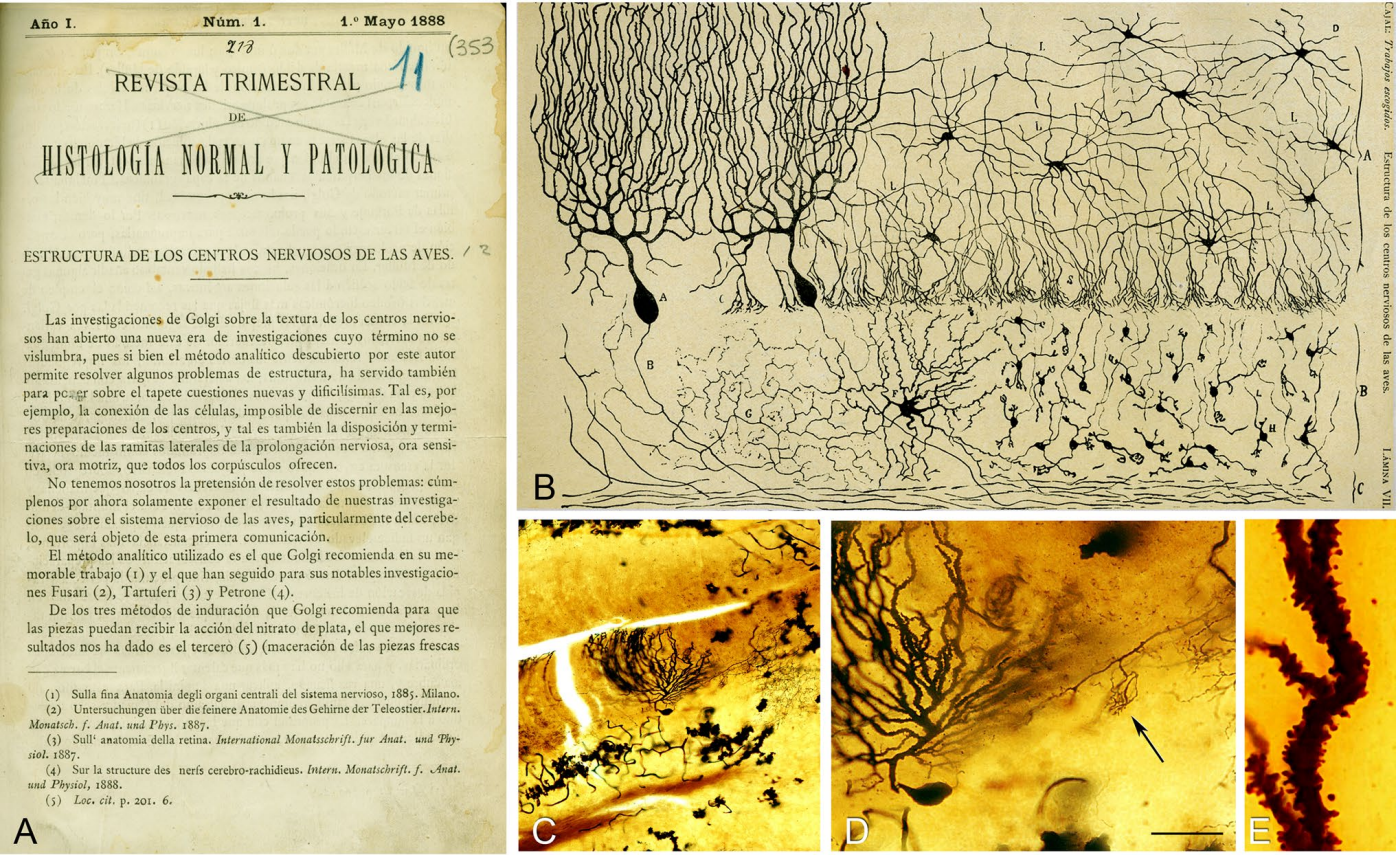
First illustration by Cajal of a Golgi-impregnated preparation of the nervous system (Cajal 1888). **A**, First page of the article and **B** illustration whose legend states: “Vertical section of a cerebellar convolution of a hen. Impregnation by the Golgi method. **A**, represents the molecular zone, **B**, designates the granular layer and **C** the white matter”. **C**, Photomicrograph from one of Cajal’s preparations of the cerebellum of an adult bird stained with the Golgi method. **D**, Higher magnification of **C** to illustrate a Purkinje cell and a basket formation (arrow). **E**, Dendrite of the Purkinje cell, which is covered by spines. In the text, Cajal said: “*…*the surface of [the dendrites of Purkinje cells] appears to be covered with thorns or short spines. (At the beginning, we thought that these eminences were the result of a tumultuous precipitation of the silver but the constancy of its existence and its presence, even in preparations in which the reaction appears to be very delicate in the remaining elements, incline us to believe this to be a normal condition)*.*” Scale bar: 200 µm in *c*; 60 µm in *d*; 8,4 µm in *e*. The histological images were obtained by Pablo García-López, Virginia García-Marín, and Miguel Freire (Legado Cajal, Instituto Cajal). Taken from DeFelipe (2010)

Following his meeting with Simarro, Cajal immediately began using the Golgi method to investigate virtually the entire nervous system. Within a year, he published his seminal work, *Estructura de los centros nerviosos de las aves* (Structure of avian nerve centers) (Cajal 1888), based on findings obtained using this technique in the avian cerebellum (Fig. 8). This study made two significant contributions. First, Cajal provided the first description of dendritic spines (a term he coined), structures that remain a focus of intense research today (DeFelipe 2015). Second, while confirming Golgi’s observation that dendrites terminate freely, Cajal crucially proposed that this principle also applies to axons and their branches.

These observations reveal another interesting aspect of the history of neuroanatomy: namely that while all scientists at the time employed similar microscopes and tissue preparation techniques, their interpretations of the microscopic world diverged significantly. The key difference between Cajal and others lay in his relentless pursuit of detail. He possessed a unique ability to notice and interpret minute structures that eluded others. A good example of this would be dendritic spines, which were initially dismissed as artifacts by some scientists, including Golgi himself (DeFelipe, 2015). Another significant example of these divergent interpretations concerns the connections between neurons: the Neuron Theory versus the Reticular Theory. These two opposing views on neuronal connectivity had significant implications for our understanding of brain function. The Reticular Theory, the predominant hypothesis at the time, postulated that nerve impulses flowed uninterrupted through a continuous network of neuronal processes. By contrast, the Neuron Theory, firmly supported by Cajal, proposed that these impulses traveled from one cell to another via specialized points of contact or contiguity, rather than continuity (Cajal, 1933). Thus, the acceptance of either hypothesis would represent a radical shift in the understanding of brain function, transitioning from the concept of a continuous neural network to that of an “infinitely fragmented” brain. There was, therefore, a clear need to investigate how the nerve impulse was transmitted from one nerve cell to another across a physical gap. This was well expressed by Cajal in *Textura* (Cajal, 1899-1904, volume 1, page 68):

Since the [dendritic and axonal processes] end freely, it must be supposed that there exists, between these [processes], a more or less intimate contact, capable of explaining the passage of [nervous] currents through a chain of conductors.

In the French version of *Histologie* (Cajal 1909-1911), the text is modified, and after “contact”, the following is added: “in a way very similar to how an electric current crosses a junction between two cables”.

An important consequence of the Neuron Theory was Cajal’s theory of the Law of Dynamic Polarization of nerve cells. At that time, nerve impulse transmission within neurons was a major conundrum. Cajal himself eloquently posed the question in *Recuerdos de mi Vida*:

In what direction does the nervous impulse travel within the neuron? Does it spread in all directions, like sound or light, or does it pass constantly in one direction like water in a watermill?

**Fig. 9:**
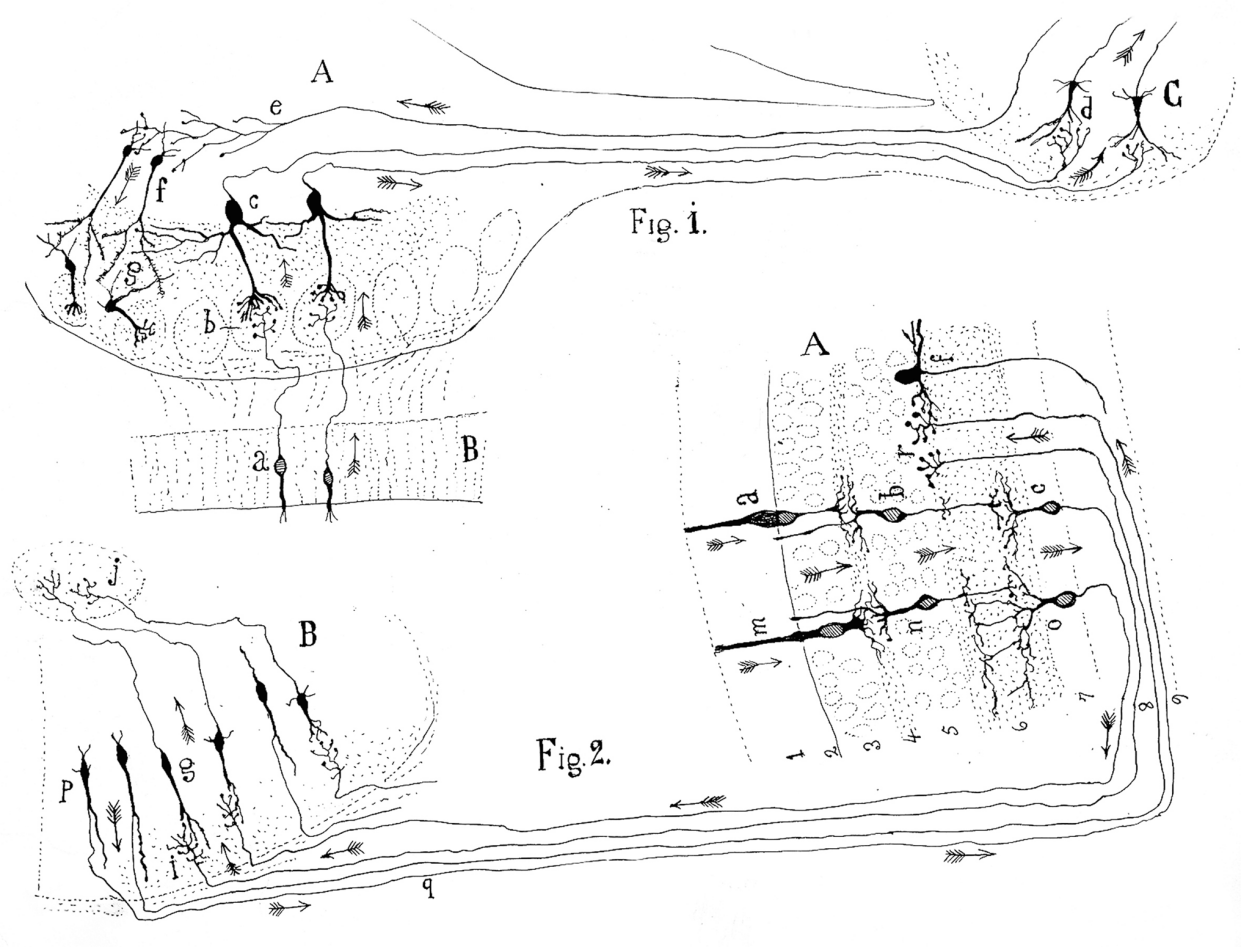
Cajal’s scheme showing the current flow in the visual and olfactory systems. This drawing was reproduced in his article *Significación fisiológica de las expansiones protoplásmicas y nerviosas de las células de la substancia gris* (Cajal 1891). The legend states: “*Fig. 1.* Scheme of cellular connections in the olfactory mucosa, olfactory bulb, tractus, and olfactory lobe of the cerebrum. The arrows indicate the direction of the currents. *A*, olfactory bulb; *B*, mucosa; *C*, olfactory lobe. *a*, *b, c, d,* one-way or centripetal pathway through which sensory or olfactory excitation passes. *e, f, g*, centrifugal pathway through which the [nervous] centers can act on the elements of the bulb, granules and nerve cells, whose protoplasmic processes penetrate the glomeruli.” “*Fig. 2.* Scheme of the visual excitation pathway through the retina, optic nerve and optic lobe of the birds. *A*, retina; *B*, optic lobe. *a, b, c*, represent a cone, a bipolar cell and a ganglion cell of the retina, respectively, the order through which visual excitation travels. *m, n, o*, parallel current emanating from the rod also involves bipolar and ganglion cells. *g*, cells of the optic lobe that receive the visual excitation and transfer it to *j*, the central ganglion. *p, q, r*, centrifugal currents that start in certain fusiform cells of the optic lobe and terminate in *r*, in the retina at the level of the spongioblasts; *f*, a spongioblast.” Arrows indicate the direction of current flow

It was widely believed that dendrites served a nourishing function, while axons transmitted nerve impulses in a cellulifugal direction—a notion largely based on the observation of cellulifugal conduction in spinal motor neuron axons. However, there was no broad consensus or clear understanding of the role of dendrites in information processing or the direction of nerve impulses in other, more complex regions of the nervous system. By 1889, Cajal recognized that dendrites, at least in some instances, were involved in receiving signals (Cajal 1889). He formalized this concept 2 years later in his Law of Dynamic Polarization (Cajal 1891). Cajal deduced this principle by observing systems like the visual and olfactory pathways, where the impulse direction was more evident (Fig. 9). He reasoned that if dendrites consistently function as receptive structures and axons as signal transmitters, then this model could be generalized to determine impulse direction throughout the central nervous system:

If in such inquiry, the [dendritic] arborization is always shown as a receptor apparatus and the [axonal arborization] as an apparatus for the *application* of the [impulses], then by analogy we would have attained a rule to judge the direction of the currents in the [nerve cells within the central nervous system].

**Fig. 10:**
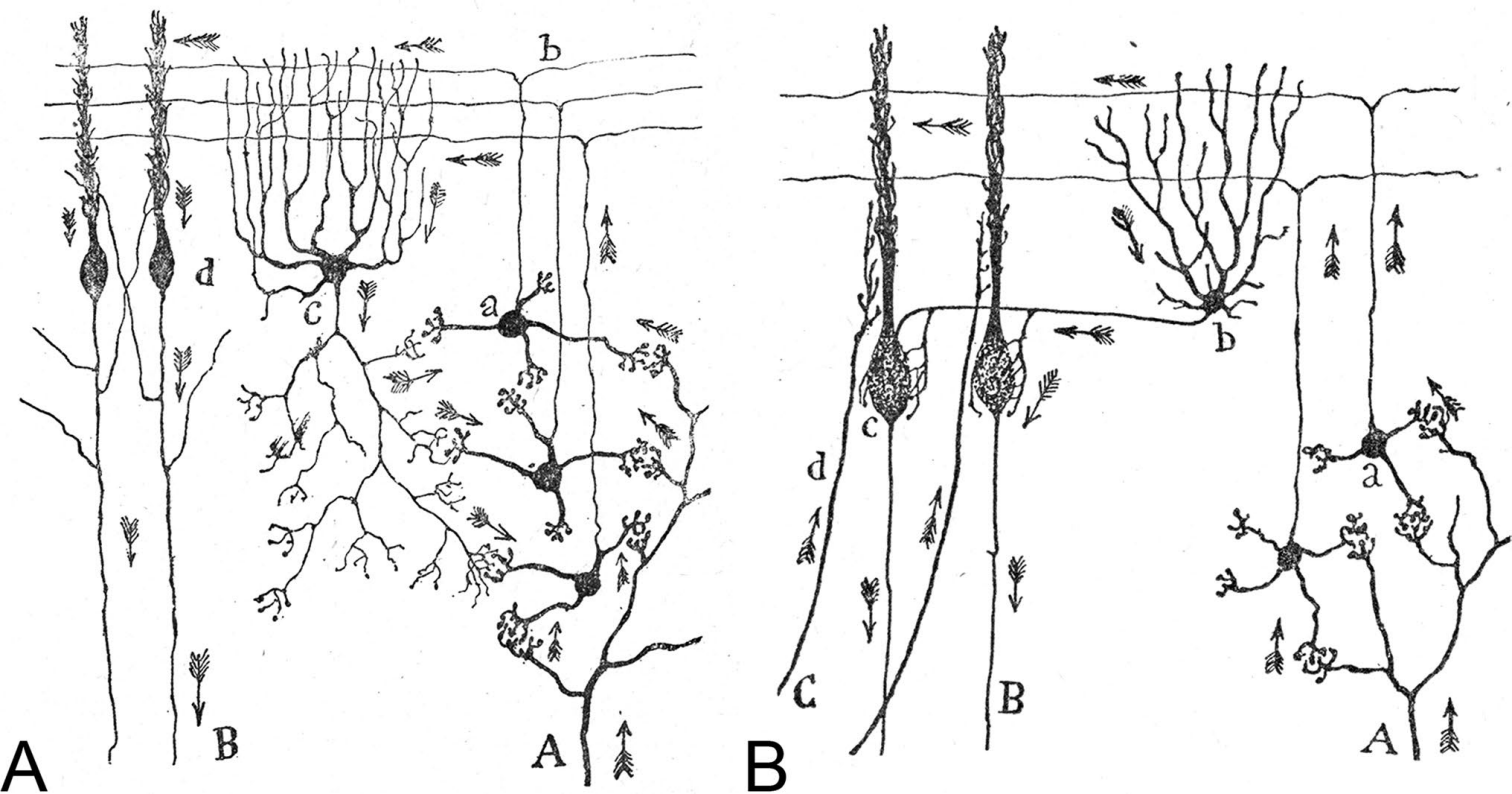
Cajal’s drawing to illustrate the participation of different types of cells in the transmission of impulses in the cerebellum based on the theory of dynamic polarization. A, The legend states: “Diagram that reveals the flow of the current contributed by the mossy fibers and the role of the Golgi cells in it. *A*, mossy fibers; *B*, Purkinje cell axons; *a*, granule cells; *b*, parallel fibers; *c*, Golgi cell; *d*, side view of Purkinje cell”. Taken from Cajal (1899– 1904; Vol. 2, 1909, Fig. 447). B, The legend states: “Diagram destined to show the participation of basket cells in the transmission of the afferent impulses. *A*, Mossy fiber; *B*, Purkinje cell axons; *C*, climbing fiber; *a*, granule cells; *b*, basket cell; *c*, [side view of] Purkinje cell”. Taken from Cajal (1899– 1904; Vol. 2, 1909, Fig. 448)

Thus, Cajal proposed that, in general, neurons could be divided into three functionally distinct regions: a receptor apparatus (formed by the dendrites and soma), an emission apparatus (the axon) and a distribution apparatus (the terminal axonal arborization). He applied this principle across all parts of the nervous system and to various types of neurons (Fig. 10).

**Fig. 11:**
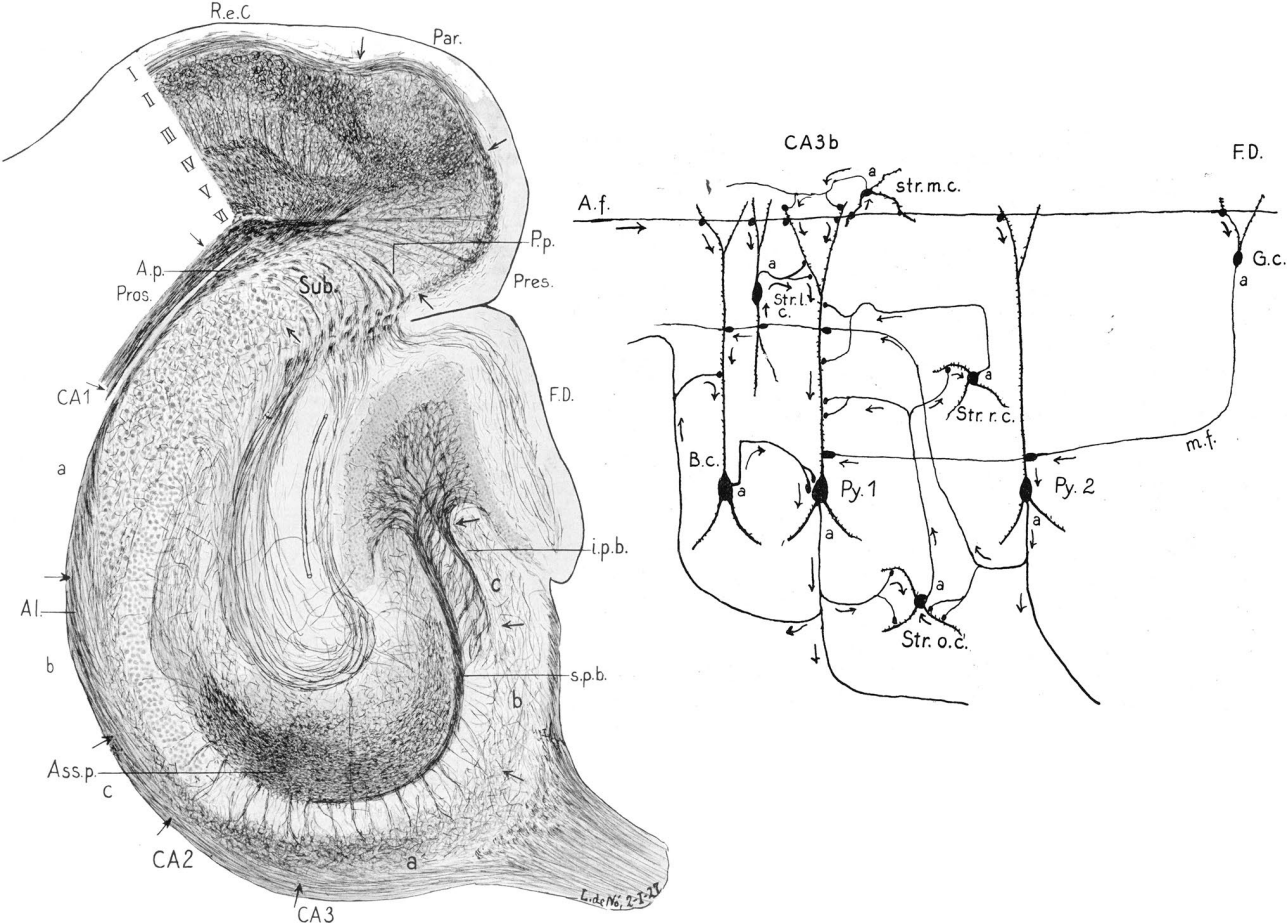
Drawing taken from Lorente de Nó (1934) to illustrate the hippocampal formation (*left*) and synaptic circuits in CA3 (*right*). *Left,* The legend states: “Horizontal section through the brain of an adult mouse. Cajal’s reduced silver method; fixation in pyridine 50%. The lettering is as in *Fig. 2* [*R.e*. Regio entorhinalis; *Aa, lib, B, C* its fields; *Par*. Parasubiculum; *Sub*. Subiculum with its two fields (*a, b*); *Pres*. Presubiculum; *Pros*. Prosubiculum with its three fields (*a, b, c*); *CA2* field CA2; *CA3* field CA3 with its three subfields (*a, b, c*); *CA4* field CA4; *F.d*. Fascia dentata; *Fi*. Fimbria; besides, *s. p. b.* and *i. p. b.* supra and infrapyramidal bundles of mossy fibers from the Fascia dentata. *A.p.* alvear path from the Area entorhinalis; *P. p.* perforant path from the area entorhinalis; *AI.* Alveus or white substance; *Ass. p.* Association (axial) path in front of *CA2*. The layers of the area entorhinalis have been marked with roman numerals (I–VI). Note the presence between III and IV of an association path, which ends in the parasubiculum *(Par.) Right*, The legend states: “Several of the many possible paths between the afferent fibres of the perforant path and the effector pyramid, *Py.* 1; field *CA 3b.* The afferent fibre *A.f.* establishes contacts with the pyramids *Py.* 1 and *Py.* 2, with the cells with short axis cylinder of the Stratum moleculare *(str. m. c.),* lacunosum *(str. l. c.)* and pyramidale (basket cell, *B. c.)* and with the granules of the Fascia dentata (g. *c.).* When these cells discharge, their impulses are transmitted to cell *Py.* 1. Besides, when cells *Py* discharge, other cells with short axis cylinder, of the Stratum radiatum *(Str. r. c.)* and oriens *(Str. o. c.),* which were not affected by the afferent impulse, are brought into activity; their impulses are again transmitted to cell *Py.* 1. The axons are marked with *a*”

Cajal’s theory of dynamic polarization was instrumental in mapping and interpreting information flow within the brain’s intricate microcircuits. For instance, Lorente de Nó, in his 1934 description of the CA3 connectivity (Fig. 11), explicitly noted: “arrows indicate the direction of transmission of the impulses according to Cajal’s law of axonal polarization. If this law is not accomplished, i.e., if the synapse is not irreversible, the interpretation of the diagram would be quite different from that proposed in the text.” This demonstrates the theory’s significance in relation to understanding the functional organization of neuronal connections.

Nevertheless, in Cajal’s own words, after 50 years of research, it was clear to him that the Neuron Theory was accurate, but other scientists were not convinced. Indeed, articles supporting the Reticular Theory were still being published in the first quarter of the twentieth century. Recognizing the ongoing debate, Cajal felt compelled to summarize his studies and ideas on the Neuron Theory while rebutting the erroneous interpretations of the reticularists. In 1933, he published a seminal article entitled *¿Neuronismo o reticularismo? Las pruebas objetivas de la unidad anatómica de las células nerviosas* in the journal *Archivos de Neurobiología*. This work was later published in the form of a book in 1952 and translated into English in 1954, under the title *Neuron Theory or Reticular Theory? Objective Evidence of the Anatomical Unity of Nerve Cells* (Cajal 1933). In this work, Cajal provided extensive explanations for why different scientists interpreted microscopic images differently when analyzing neuronal connections using the Golgi method or other techniques. This publication exemplifies Cajal’s analytical skills and logical reasoning in defending the Neuron Theory.

## Acknowledging Cajal’s scientific achievements

The observations, concepts, and theories of Cajal published between 1888 and 1903 following his meeting with Simarro soon had a profound impact on researchers of the era. Cajal began to be known and admired by the international scientific community after his participation in the Congress of the German Society of Anatomy, held at the University of Berlin in October 1889.

According to Cajal’s own account in *Recuerdos de mi vida* (Cajal, 1917, page 145), the congress had a demonstration room equipped with various microscopes. Cajal described how there he used two or three magnifying instruments, in addition to his “excellent Zeiss model, brought just in case”, to show his preparations of the cerebellum, retina, and spinal cord. Among the most distinguished histologists and anatomists of the time who showed great interest in his preparations were His, Schwalbe, Retzius, Waldeyer, and, most notably, Kölliker—one of the most influential scientists of the time. Kölliker was so impressed with Cajal’s discoveries that he told him (*Recuerdos de mi vida*, Cajal, 1917, page 147):

The results that you have obtained are so beautiful that I am planning to immediately undertake a series of confirmatory studies by adopting your methodology. I have discovered you, and I wish to make my discovery known in Germany.

This historical milestone was recorded by Professor Arthur van Gehuchten (1861–1914), one of those present at the event, in his response speech to the tribute received on the occasion of his 25 years of teaching at the University of Leuven (van Gehuchten 1913). Cajal’s predicament is vividly portrayed in the paragraph where van Gehuchten recounts this event (pages 32–33):

The facts described [by Cajal] in his first publications were so strange that the histologists of the time […] received them with the greatest skepticism. The distrust was such that, at the anatomical congress held in Berlin in 1889, Cajal, who afterward became the great histologist of Madrid, found himself alone, provoking around him only smiles of incredulity […] I can still see him taking aside Kölliker, who was then the unquestioned master of German histology, and dragging him into a corner of the demonstration hall to show him under the microscope his admirable preparations, and to convince him at the same time of the reality of the facts which he claimed to have discovered. This demonstration was so decisive that a few months later the Würzbourg histologist [Kölliker] confirmed all the facts stated by Cajal.

**Fig. 12:**
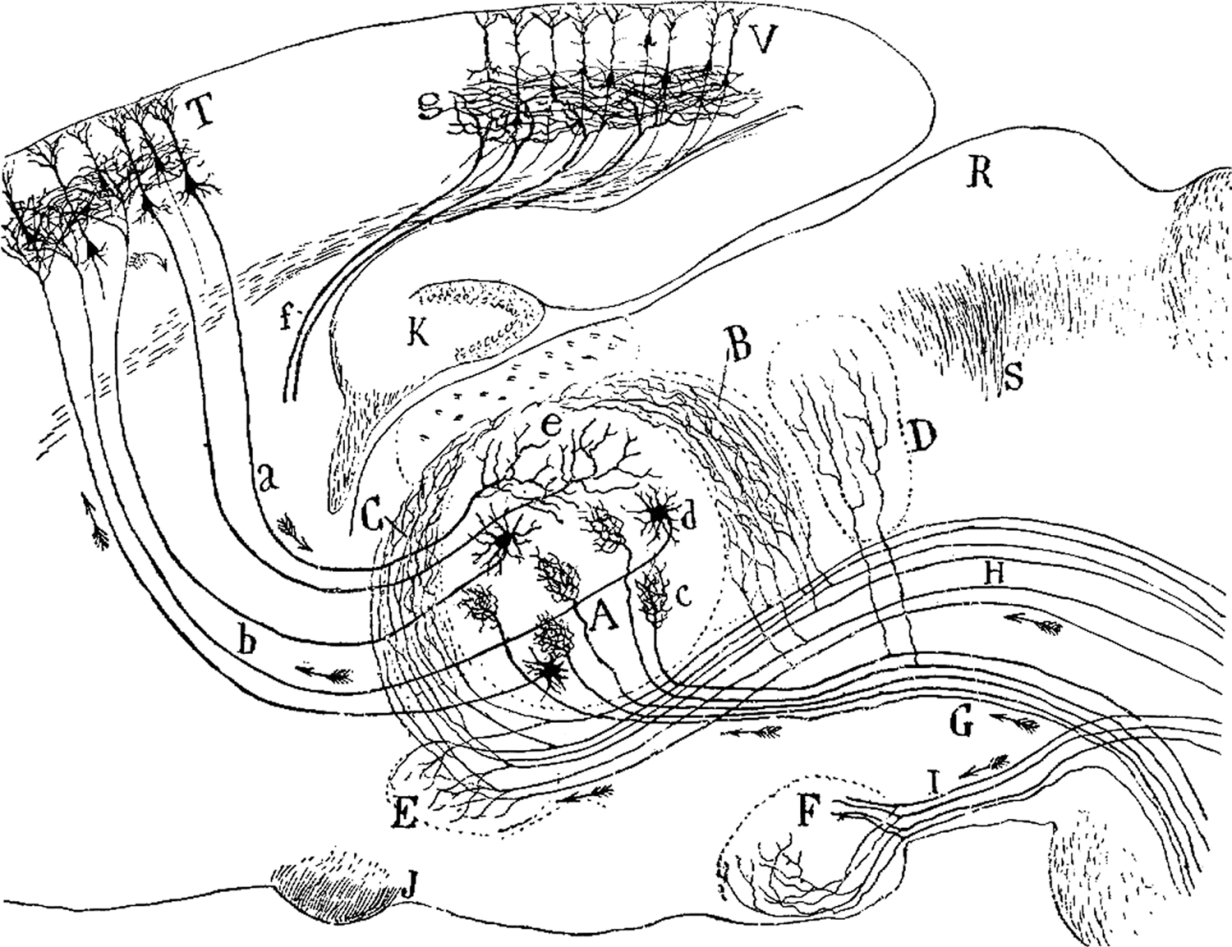
Cajal’s drawing showing the connections between different brain regions. The figure legend states: “Schematic figure of the sensory pathways (lemniscus and trigeminal pathway) showing the components of the dorsal thalamus. *A*, sensory nucleus of the thalamus; *B*, *C*, accessory or trigeminal sensory nuclei; *D*, posterior nucleus of the thalamus; *E*, nucleus of the *zona incerta*; *F*, lateral mammillary nucleus; *G*, medial lemniscus; *H*, central pathways of the fifth nerve and of other origins; *I*, peduncle of the mammillary body; *J*, optic chiasm; *K*, hippocampal gyrus; *T*, motor cerebral cortex; *V*, visual cerebral cortex; *a*, corticothalamic fibers; *b*, thalamocortical sensory pathway; *f*, thalamocortical visual pathway; *R*, branchium of the superior colliculus; *S*, posterior commissure; *c*, terminal arborizations of lemniscal fibers; *d*, long-axon neuron of the sensory nucleus; *g*, terminal plexus of optic radiation fibers”. Taken from Cajal (1903; *Fig. 1*)

Kölliker disseminated Cajal’s observations, concepts, and theories, which had a rapid and profound influence on researchers of his time. As a result of this recognition, distinguished institutions and researchers invited Cajal to expound his findings, discussing the morphological aspects of his studies and, importantly, emphasizing the functional implications for neuronal circuitry. In several of these illustrations, he traced how axons originating from different functional regions of the brain connect with neurons in specific areas (illustrating the multifunctional integration of inputs from various sources) and revealed how these neurons distribute the ‘processed’ information within the region and to other parts of the brain (Fig. 12). During this period, Cajal skillfully described the micro-organization of almost every region of the central nervous system, and the results were later summarized in his classic book *Textura del sistema nervioso del hombre y de los vertebrados* (Cajal, 1899–1904). This text was more widely disseminated through a French translation (Cajal 1909–1911) which is still widely used as a reference book.

**Fig. 13:**
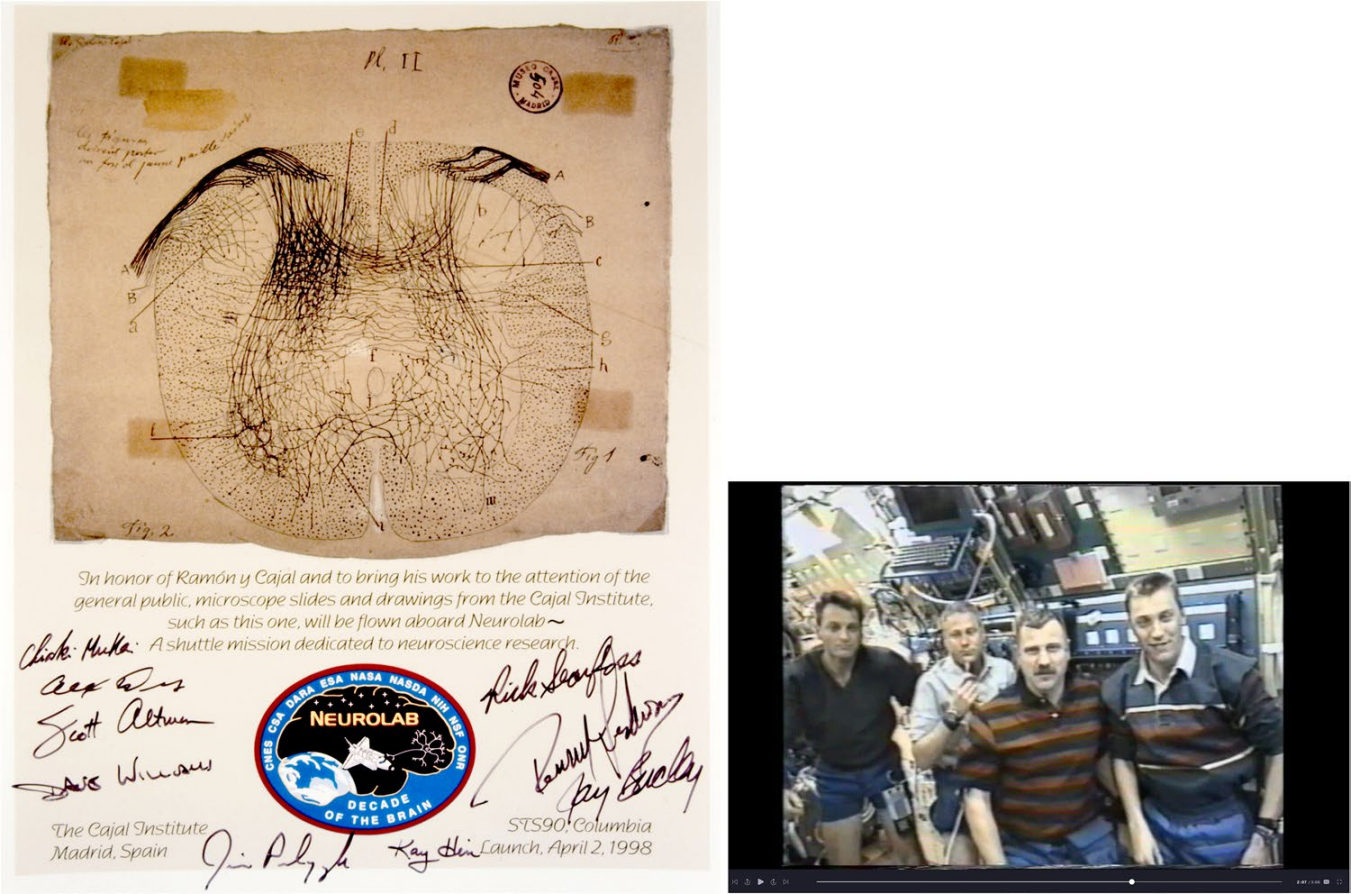
*Left*: A poster commemorating the work of Cajal, featuring a reproduction of one of the nine original drawings by Cajal that were exhibited in the NASA museum during the mission to educate the public about his contributions to neuroscience. The signatures belong to the crew members. *Right*: A screenshot from a video clip recorded during the Neurolab mission, capturing the moment when crew members—(from left to right) Richard Linnehan, Jay Buckey, Dave Williams, and James Pawelczyk—delivered a televised lecture to multiple countries (Supplementary Video). During the lecture, they highlighted how the Neurolab mission carried a piece of neuroscience history to honor Cajal: twelve original histological preparations made by Cajal himself. The astronauts further paid tribute to Cajal by emphasizing the importance of his contributions to brain research, particularly Neuron Theory and neuroplasticity, and their relevance to the Neurolab mission. Video courtesy of NASA. Neurolab (STS-90) was a NASA research mission primarily focused on studying how the nervous system responds to microgravity— an essential question for future long-duration spaceflights. The crew conducted various experiments involving animals—including rats, mice, fish, snails, and crickets—aboard the Space Shuttle Columbia, while also serving as human subjects themselves in a series of sophisticated biomedical examinations (Buckey and Homick, 2003). The shuttle was launched on April 17 and landed on May 4, 1998, at Kennedy Space Center in Cape Canaveral, Florida, USA

Cajal also received numerous awards and distinctions, including some of the most prestigious awards of his time: the Moscow Award (1900); the Helmholt gold medal (1905); and the Nobel Prize for Physiology or Medicine (1906) that he shared with Golgi. Over the years, Cajal has been honored with numerous tributes, including a homage during NASA’s Neurolab space flight mission in 1998 (Buckey and Homick 2003). Figure 13 presents one of the posters commemorating the work of Cajal, signed by the crew members of the Neurolab mission, along with a screenshot from a video taken during the flight. The video captures the moment when the astronauts paid tribute to Cajal, highlighting the significance of his studies.

**Fig. 14:**
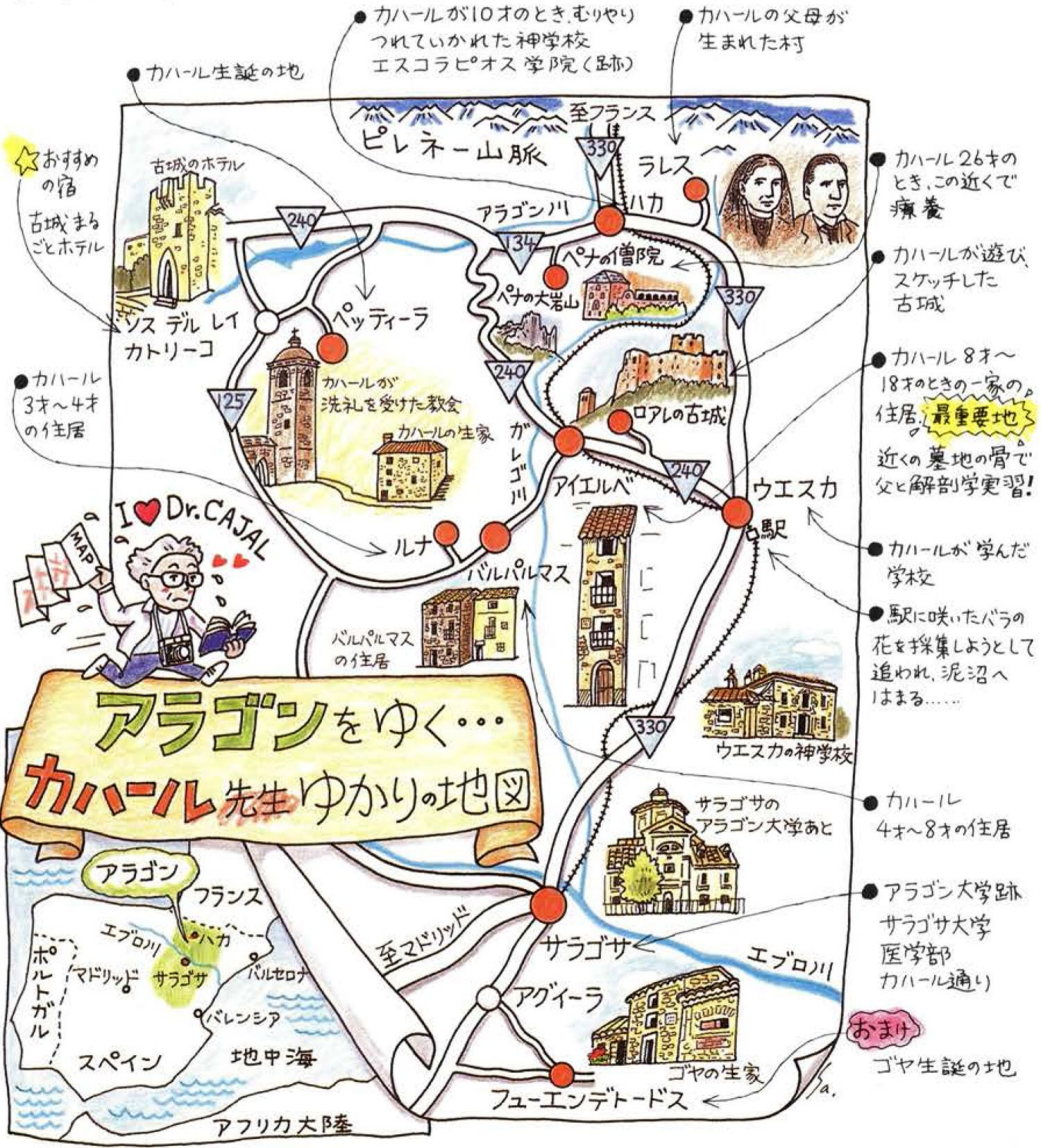
Cartoon map created by Professor Fusahiro Ikuta in 1996 to illustrate Cajal’s birthplace (Petilla de Aragón) and other significant locations related to his life (see text for details)

One of the most remarkable international meetings held in his honor was in 2005, in Petilla de Aragón, a small village in Navarra (Northern Spain), where Cajal was born. The attending scientists hailed from around the world and included researchers from Japan, which brings to mind the cartoon map (Fig. 14) by Professor Fusahiro Ikuta (Ikuta 1996) that I presented at that meeting. This map is a delightful blend of geography, illustration, and storytelling, offering an overview of Cajal’s life and the places that were significant to him, such as his birthplace. Since reaching Petilla de Aragón is somewhat complicated, I sent this map in jest to some of my colleagues who asked me for directions to help them find their way!

Despite the fact that hundreds of studies have been carried out since the times of Cajal, there are still no universally accepted scientific criteria to define the fundamental morphological, molecular, and physiological characteristics that distinguish the various types of cortical neurons. In this regard, it is important to acknowledge Golgi not only for being the discoverer of the method that bears his name, but also for his significant early contributions to the study of the nervous system (Bentivoglio et al. 2019). For instance, Golgi was the first to propose that, in general, neurons could be classified into two morphologically and physiologically distinct types: *motor (type I)* and *sensory (type II)*. Motor neurons had long axons that gave rise to collaterals, with the main axon leaving the gray matter (projection neurons). By contrast, sensory neurons had short axons that arborized near the parent cell and remained within the gray matter (intrinsic neurons). The former were considered motor neurons because their axons were thought to be continuous with motor roots, while the latter were classified as sensory neurons because their axonal branches were linked with afferent fibers. However, according to Cajal (1892), it was not physiologically feasible to maintain Golgi’s distinction between these two morphological types. For example, retinal ganglion cells are sensory neurons, yet they have long axons (i.e., they are projecting neurons). Consequently, Cajal (1891, 1892) redefined Golgi’s classification, referring to neurons simply as *long-axon cells* and *short-axon cells*, without making assumptions about their physiological roles. Since then, the term “short-axon cell” has commonly been used synonymously with *interneurons* (DeFelipe 2002b). The 2005 meeting in Petilla, which was rooted in the early studies of Cajal and Golgi, led to a series of fundamental agreements that serve as a framework for the scientific community regarding the nomenclature of cortical interneurons, known as Petilla Terminology (Ascoli et al. 2008). I believe that Golgi and Cajal would be proud of the enduring impact of their research, despite their divergent views.

## Supplementary Information

Below is the link to the electronic supplementary material.

Supplementary Video (MP4 359537 kb)
